# Inflammation in Human Heart Failure: Major Mediators and Therapeutic Targets

**DOI:** 10.3389/fphys.2021.746494

**Published:** 2021-10-11

**Authors:** Marta Reina-Couto, Patrícia Pereira-Terra, Janete Quelhas-Santos, Carolina Silva-Pereira, António Albino-Teixeira, Teresa Sousa

**Affiliations:** ^1^Departamento de Biomedicina – Unidade de Farmacologia e Terapêutica, Faculdade de Medicina, Universidade do Porto, Porto, Portugal; ^2^Centro de Investigação Farmacológica e Inovação Medicamentosa, Universidade do Porto (MedInUP), Porto, Portugal; ^3^Departamento de Medicina Intensiva, Centro Hospitalar e Universitário São João, Porto, Portugal

**Keywords:** inflammation, chronic heart failure (CHF), acute heart failure (AHF), cardiogenic shock (CS), inflammatory mediators, clinical trials, anti-inflammatory strategies

## Abstract

Inflammation has been recognized as a major pathophysiological contributor to the entire spectrum of human heart failure (HF), including HF with reduced ejection fraction, HF with preserved ejection fraction, acute HF and cardiogenic shock. Nevertheless, the results of several trials attempting anti-inflammatory strategies in HF patients have not been consistent or motivating and the clinical implementation of anti-inflammatory treatments for HF still requires larger and longer trials, as well as novel and/or more specific drugs. The present work reviews the different inflammatory mechanisms contributing to each type of HF, the major inflammatory mediators involved, namely tumor necrosis factor alpha, the interleukins 1, 6, 8, 10, 18, and 33, C-reactive protein and the enzymes myeloperoxidase and inducible nitric oxide synthase, and their effects on heart function. Furthermore, several trials targeting these mediators or involving other anti-inflammatory treatments in human HF are also described and analyzed. Future therapeutic advances will likely involve tailored anti-inflammatory treatments according to the patient’s inflammatory profile, as well as the development of resolution pharmacology aimed at stimulating resolution of inflammation pathways in HF.

## Introduction

Heart failure (HF) is a complex clinical syndrome caused by structural and/or functional cardiac abnormalities which result in the impairment of ventricular filling and/or ejection (Ponikowski et al., 2016). Although hemodynamic and neurohormonal counterregulatory responses are activated in order to maintain adequate tissue perfusion, their perpetuation contributes to structural and functional damage at medium and long term (Gullestad et al., 2012; Kemp and Conte, 2012; Braunwald, 2013). Chronic HF (CHF) is currently divided in three categories based on left ventricular ejection fraction (LVEF), namely reduced (HFrEF), preserved (HFpEF) or mid-range (HFmrEF) (Ponikowski et al., 2016). This last category (HFmrEF) (Castillo et al., 2020), recently individualized in European guidelines, represents an intermediate range of LVEF that has been less rigorously studied, with clinical characteristics that resemble those of the HFpEF group (Hsu et al., 2017) but with a higher prevalence of coronary artery disease, and so a similar response to the treatment of HFrEF patients (Tomasoni et al., 2019).

Despite therapeutic advances, CHF inexorably progresses with acute episodes, requiring recurrently urgent hospitalization and medical support (Ramani et al., 2010; Ponikowski et al., 2016). Although acute heart failure (AHF) frequently refers to this acute decompensation of CHF, it may also correspond to the new-onset form (“*de novo* HF”) caused by direct ischemic, infectious/inflammatory or toxic insults to the myocardium, or indirectly by mechanical development of acute valve insufficiency or cardiac tamponade (Spodick, 2003; Ponikowski et al., 2016). Cardiogenic shock (CS), the severest form of these AHF syndromes, evolve as a continuum from those underlying precipitants until the subsequent state of low cardiac output, organ failure and death (Shpektor, 2010; Mebazaa et al., 2016; van Diepen et al., 2017; Chioncel et al., 2020). Therefore, there is an urgent need to explore new pathophysiological pathways and therapeutic strategies for HF (Yndestad et al., 2006; Heymans et al., 2009; Desai and Stevenson, 2012).

Inflammation is accepted as an important pathophysiological factor in both AHF and CHF, predicting poor prognosis independently of LVEF (Murphy et al., 2020), although it appears to contribute in different ways to each type of HF (Castillo et al., 2020; Murphy et al., 2020; Srivastava et al., 2020).

In HFpEF, where a more evident association with inflammatory markers is reported, underlying comorbidities, such as obesity, arterial hypertension, diabetes, chronic obstructive pulmonary disease, chronic kidney disease and also aging, trigger a systemic inflammatory state which cause microvascular endothelial cell inflammation associated with increased reactive oxygen species (ROS) production and decreased nitric oxide (NO) bioavailability (Paulus and Tschope, 2013). Chronic inflammation also favors the infiltration of monocytes into the myocardium and further differentiation into proinflammatory macrophages (M1) (Glezeva and Baugh, 2014). These events promote adverse left ventricle remodeling and relaxation impairment (Glezeva and Baugh, 2014).

In HFrEF, myocardial injury is the main trigger for the inflammatory responses mediated by the innate and adaptive immune systems (Van Linthout and Tschope, 2017; Adamo et al., 2020). These are characterized by an initial increase in proinflammatory cytokines and chemokines, along with the infiltration of neutrophils and monocytes into the injured myocardium (Adamo et al., 2020). Subsequently, in the resolution and repair phase (also termed “proliferative”), there is the phagocytosis of apoptotic and necrotic cells, the influx of adaptive immune cells (T-and B-cells), the activation of collagen-synthesizing myofibroblasts and the production of anti-inflammatory and proresolving molecules (e.g., transforming growth factor beta, lipoxins, IL-10) (Adamo et al., 2020). The maturation phase is marked by the apoptosis of reparative cells and scar maturation (Adamo et al., 2020). The maintenance of a chronic inflammatory status may result from sustained myocardial injury and continuous activation of proinflammatory cascades (Reina-Couto et al., 2016; Adamo et al., 2020). Importantly, activation of classic neurohormonal systems, such as the sympathetic nervous system and the renin-angiotensin-aldosterone system and hemodynamic overload can also trigger sustained myocardial inflammatory responses – termed as para-inflammation – even in the absence of tissue injury (Adamo et al., 2020). The persistence of inflammation may also be caused by a failure in the resolution of inflammation mechanisms (Reina-Couto et al., 2016). Of note, we and others have observed that CHF is associated with impaired resolution of inflammation, namely decreased plasma and urinary lipoxins in CHF patients with severe New York Heart Association (NYHA) functional classes compared to CHF patients with mild-to-moderate NYHA classes, and significantly lower plasma concentration of resolvin D1 in patients with CHF compared to healthy age-matched controls (Reina-Couto et al., 2014; Chiurchiu et al., 2019). However, so far, targeting of resolution of inflammation pathways has only been attempted in experimental HF models (Reina-Couto et al., 2016; Kain et al., 2017; Halade et al., 2018).

In AHF, the inflammatory response may arise from antigenic stimulation during infection (e.g., viral myocarditis) or as a result of hemodynamic stress (Chen et al., 2008; Virzi et al., 2018). Systemic congestion and/or peripheral hypoperfusion cause neurohormonal activation, inflammation and oxidative stress which damage endothelial glycocalix and consequently impair endothelial function and fluid homeostasis (Harjola et al., 2017). Inflammatory activation also promotes a prothrombotic and proapoptotic environment (Mentz and O’Connor, 2016). Noteworthy, neurohormonal and inflammatory activation seem to persist beyond the acute event and may contribute to the high rehospitalization rates of this syndrome (Cotter et al., 2008; Reina-Couto et al., 2020). In CS, low cardiac output leads to systemic hypoperfusion caused by or followed by a systemic inflammatory response syndrome and end-organ injury that, if not promptly treated or supported, leads to death (Shpektor, 2010; van Diepen et al., 2017; Chioncel et al., 2020; Cuinet et al., 2020). Very early after CS onset, an inflammatory environment seen in 40% of the CS patients can contribute to the CS hallmark vasodilation (Kohsaka et al., 2005), with concentrations of various interleukins being associated with mortality (Prondzinsky et al., 2012). This marked inflammatory activation may be due to direct myocardial necrosis and tissue ischemia, inducing the release of damage-associated molecular patterns (DAMPs), mostly recognized by Toll-like receptor 4, highly expressed in the heart, that contributes to myocardial inflammation that occurs in HF (Yang et al., 2016). Also indirect but early secondary hypoperfusion leading to intestinal ischemia may promote the translocation of bacteria and pathogen-associated molecular patterns (PAMPs) (Shpektor, 2010; Cuinet et al., 2020) and, alongside with the release of inflammatory mediators by the spleen or adipose tissue and the para-inflammation generated by the comorbidities and endothelial dysfunction, contribute to cardiac deterioration. The intensity of this inflammatory response in CS patients is associated with CS severity (Geppert et al., 2006).

## Major Inflammatory Mediators in Heart Failure

### Tumor Necrosis Factor Alpha

Tumor necrosis factor alpha (TNF-α) is the most studied proinflammatory cytokine in HF (Hanna and Frangogiannis, 2020). In the heart, it can be produced by different types of cells such as activated macrophages, cardiomyocytes, vascular cells and mast cells (Urschel and Cicha, 2015; Hanna and Frangogiannis, 2020). TNF-α exists in membrane-bound or cytosolic forms and exerts its actions by binding to cell membrane TNFR1 or TNFR2 receptors (Urschel and Cicha, 2015; Bartekova et al., 2018). TNF-α has been shown to mediate several adverse effects on heart function and structure, namely negative inotropic actions due to the disruption of calcium homeostasis, upregulation of other inflammatory molecules, including induction of inducible NO synthase, enhancement of oxidative stress and consequent mitochondrial DNA damage, promotion of apoptosis and extracellular matrix degradation and increase of microvascular endothelial permeability and activation, enhancing endothelial-leukocyte interactions (Mattila et al., 1992; Yokoyama et al., 1993; Li et al., 2000; Sanders et al., 2001; Sivasubramanian et al., 2001; Suematsu et al., 2003; Haudek et al., 2007; Hamid et al., 2009; Urschel and Cicha, 2015). Increased TNF-α concentrations have been detected in patients with HFrEF, HFpEF, patients with AHF and CS (Levine et al., 1990; Tentolouris et al., 2004; Debrunner et al., 2008; Putko et al., 2014; Pugliese et al., 2020). Despite large evidence of the protective effects of TNF-α inhibition or deletion in experimental models of HF (Berry et al., 2004; Moe et al., 2004; Sun et al., 2007; Jobe et al., 2009), studies in HF patients have been disappointing (Chung et al., 2003; Mann et al., 2004). It has been suggested that TNF-α may exert not only deleterious, but also protective effects, which may be compromised under high intensity TNF-α blockade (Hanna and Frangogiannis, 2020; Murphy et al., 2020). Of note, in experimental cardiac ischemia-reperfusion injury, low doses of TNF-α were shown to be cardioprotective, while higher doses had deleterious cardiac effects (Rathi et al., 2002; Saini et al., 2005). Dose-dependent effects of TNF-α may difficult patient treatment since an optimal amount of this cytokine may be required to counteract excessive remodeling and preserve cardiac function (Hanna and Frangogiannis, 2020).

### Interleukin 1

Interleukin 1 (IL-1) seems to be a major mediator of inflammation in heart diseases (Szekely and Arbel, 2018; Hanna and Frangogiannis, 2020). Among the 11 ligands of the IL-1 family, the best known members acting on the cardiovascular system are IL-1α, IL-1β, IL-18, and IL-33 (Hanna and Frangogiannis, 2020). Binding of IL-1α or IL-1β to the IL-1 type 1 receptor (IL-1R1) initiates inflammatory responses, while the IL-1 receptor type 2 (IL-1R2) functions as a decoy receptor and does not initiate signal transduction. The IL-1 receptor type 3 (IL-1R3) acts as a co-receptor for IL-1R1, being responsible for signaling after the binding of IL-1α or IL-1β to IL-1R1 (Kaneko et al., 2019). The generation of the active form of IL-1β from its precursor protein is primarily dependent on the enzymatic activity of caspase-1, which in turn is activated by the NOD-like receptor family, pyrin domain containing 3 (NLRP3) inflammasome, a macromolecular structure containing the intracellular sensing protein NLRP3 that responds to danger-associated signals (Frangogiannis, 2015; Abbate et al., 2020). The activation of the NRLP3 inflammasome in cardiac fibroblasts and cardiomyocytes is induced by myocardial injury and may contribute to the post-infarction inflammatory process that aggravates cardiac injury (Frangogiannis, 2015; Segiet et al., 2019a).

In HF, IL-1 can be produced by immune cells, cardiomyocytes, vascular cells and fibroblasts (Hanna and Frangogiannis, 2020). IL-1 contributes to cardiac dysfunction and remodeling by reducing beta-adrenergic responsiveness of L-type calcium channels and the expression of genes involved in the regulation of calcium homeostasis, by stimulating apoptosis in cardiomyocytes, by inducing the activation of leukocytes and endothelial cells, thus promoting their interaction and increasing the recruitment of inflammatory cells to the myocardium, by favoring fibrosis and by promoting arterial stiffness and microvascular inflammation (Szekely and Arbel, 2018; Hanna and Frangogiannis, 2020). IL-1β also decreases energy production and myocardial contractility by directly damaging mitochondria (Zell et al., 1997; Szekely and Arbel, 2018).

### Interleukin 6

The proinflammatory cytokine interleukin 6 (IL-6) is an important player in the acute phase response of inflammation, being a stimulus for C-reactive protein production by the liver, but also appears to contribute to the transition from acute to chronic inflammation by favoring the change from neutrophil to monocyte recruitment (Gabay, 2006; Huynh et al., 2015; Hanna and Frangogiannis, 2020). IL-6 is produced by several cell types like macrophages, T-lymphocytes, endothelial cells, cardiomyocytes and fibroblasts (Mihara et al., 2012; Hanna and Frangogiannis, 2020). Although both proinflammatory and anti-inflammatory effects have been described for this cytokine, its effects in HF appear to be mostly proinflammatory (Bartekova et al., 2018; Hanna and Frangogiannis, 2020).

IL-6 signaling classically involves cytokine binding to the cell surface IL-6 receptor and further association of the cytokine-receptor complex with gp130, dimerization and signaling initiation (Hanna and Frangogiannis, 2020). However, IL-6 receptor can be cleaved by proteases, originating a soluble form that can bind IL-6 and initiate signaling in cells that do not express this receptor, thus increasing the complexity of IL-6 cellular effects (Hanna and Frangogiannis, 2020). Regarding the heart, IL-6 has been shown to exert negative inotropic effects and to promote hypertrophy and fibrosis, contributing to increased myocardial stiffness (Hanna and Frangogiannis, 2020). The impairment of myocardial contractility appears to be related with the upregulation of myocardial NO synthase and downregulation of the sarcoplasmic reticulum Ca^2+^ ATPase (SERCA2) (Finkel et al., 1992; Villegas et al., 2000). IL-6 has also a negative impact on renal function since it activates the epithelial sodium channel (ENaC) in the distal tubule, impairing natriuresis (Li et al., 2010). Of note, high values of IL-6 are associated with diuretic resistance in HF patients (Kielar et al., 2005; Zhang W. et al., 2012).

Higher IL-6 concentrations have been associated with worse clinical status in CHF patients, being an independent predictor of mortality (Tsutamoto et al., 1998; Maeda et al., 2000). In AHF, IL-6 values at 48–72 h were found to be independently associated with 30-day mortality, but not with 180-day mortality (Perez et al., 2021). CS patients also exhibit an early rise of IL-6 which correlates with the severity of circulatory failure (Cuinet et al., 2020).

### Interleukin-8

Interleukin-8 (IL-8), also termed CXCL8, is a chemokine produced by monocytes, macrophages, neutrophils, epithelial cells, fibroblasts, smooth muscle cells, and endothelial cells, being induced by diverse stimuli such as shear stress, ischemia and hypoxia (Bartekova et al., 2018; Segiet et al., 2019a). It is highly expressed in atherosclerotic lesion macrophages. IL-8 induces the firm adhesion of monocytes in the initial stages of atherogenesis (Apostolakis et al., 2009). In the heart after infarction, it appears to mediate neutrophil activation and chemotaxis, as well as angiogenesis (Apostolakis et al., 2009; Bartekova et al., 2018). IL-8 is increased in CHF, being related with worse outcomes (Damas et al., 2000; Nymo et al., 2014). It also appears to predict HF development after myocardial infarction (Dominguez-Rodriguez et al., 2006; De Gennaro et al., 2012). Increased IL-8 values have also been observed in AHF and CS patients (Prondzinsky et al., 2012; Husebye et al., 2014). An important feature of IL-8 is its relative longevity in acute inflammatory conditions, remaining active for a prolonged period, in contrast to other inflammatory cytokines (Apostolakis et al., 2009). It is not clear whether IL-8 contributes to cardiac injury or is cardioprotective in the post-infarcted heart through its angiogenic effects (Bartekova et al., 2018).

### Interleukin 10

The anti-inflammatory cytokine interleukin 10 (IL-10) is produced by several cell types, such as monocytes, macrophages, activated T and B cells, eosinophils, mast cells, dendritic cells, epithelial cells and also tumor cells (Bartekova et al., 2018; Segiet et al., 2019a). It exerts its actions by binding to a specific receptor complex, that consists of two IL-10 receptor-1 (IL-10R1) proteins and two IL-10 receptor-2 (IL-10R2) proteins (Bartekova et al., 2018). Major effects of IL-10 include the inhibition of proinflammatory cytokines secretion, reduction of NO generation and inhibition of ROS production and TNF-α-mediated oxidative stress (Kaur et al., 2009). It also appears to counteract TNF-α-induced cardiomyocyte apoptosis (Dhingra et al., 2009, 2011). In CHF patients, IL-10 values were shown to be either increased when compared to healthy controls and positively correlated with NYHA class, or unchanged (Gullestad et al., 2001b; Dixon et al., 2011). Of note, although β-adrenergic receptor stimulation has been shown to increase IL-10 production, this anti-inflammatory response appears to be reduced in moderate and severe CHF patients (Ng and Toews, 2016).

Among patients with AHF, IL-10 values did not differ when compared to healthy controls or when patients were stratified according to the presence of renal dysfunction and/or congestion (Pugliese et al., 2020). However, IL-10 values have been shown to be significantly increased and to predict outcomes in CS patients (Prondzinsky et al., 2012; Reina-Couto et al., 2019b; Cuinet et al., 2020).

### Interleukin 18

The proinflammatory cytokine interleukin 18 (IL-18) is a member of the IL-1 superfamily, being activated similarly to IL-1β by caspase-1 after inflammasome formation (Dinarello et al., 2013; O’Brien et al., 2014; Segiet et al., 2019a). IL-18 exists mostly as a soluble cytokine, although a membrane-bound form can also be found in a subset of macrophages, which can release soluble IL-18 upon stimulation with lipopolysaccharide (O’Brien et al., 2014). The receptor for IL-18 (IL-18R) is a dimer formed by the IL-18Rα chain, which is the low-affinity binding site for mature IL-18, and the IL-18R β chain, which binds the IL-18/IL-18Rα complex, thus forming a high-affinity complex that initiates signaling. These receptor subunits are members of the IL-1R family (Dinarello et al., 2013; O’Brien et al., 2014). Of note, the activation of the IL-18R requires higher amounts of IL-18 (10–20 ng/mL or higher) than the activation of IL-1R by IL-1α or IL1β (frequently activated in the pg/mL range) (Dinarello et al., 2013). The activity of IL-18 is negatively regulated by IL-18 binding protein (IL-18BP), which is constitutively secreted and binds IL-18 with extremely high affinity (Dinarello et al., 2013). Since IL-18BP neutralizes the circulating IL-18, the concentration of the free form of IL-18 is lower than the total circulating IL-18 amount. Moreover, the values of free IL-18 appear to have a better correlation with disease activity than total IL-18 concentration (Dinarello et al., 2013; O’Brien et al., 2014; Kaneko et al., 2019).

In the heart, IL-18 induces an inflammatory response by increasing IFN-γ production by infiltrated neutrophils, resident macrophages and endothelial cells and by stimulating IL-1β and TNF-α generation by endothelial cells. IL-18 also increases the expression of vascular cell adhesion molecule 1 (VCAM-1) and intercellular adhesion molecule 1 (ICAM-1) in endothelial cells and cardiomyocytes, thus increasing leukocyte recruitment into the injured myocardium and amplifying cardiac inflammation (Wang et al., 2008). IL-18 has also been shown to induce cardiac hypertrophy and fibrosis, apoptosis, contractile dysfunction and decreased β-adrenergic receptor responsiveness (Wang et al., 2008; O’Brien et al., 2014; Segiet et al., 2019a). Some of these effects appear to be mediated, at least in part, by the induction of other cytokines and chemokines, such as IL-1β, TNF-α, and IFN-γ (Wang et al., 2008; O’Brien et al., 2014).

The precursor form of IL-18 is constitutively expressed in several cell types such as monocytes, macrophages, epithelial cells, endothelial cells and cardiomyocytes (Dinarello et al., 2013; O’Brien et al., 2014; Segiet et al., 2019a). Following acute MI, the activation of the inflammasome in leukocytes, fibroblasts and cardiomyocytes in the ischemic myocardial tissue increases the local production of IL-18 (O’Brien et al., 2014). In the human failing ischemic myocardium, IL-18 was detected in endothelial cells, macrophages and cardiomyocytes (Mallat et al., 2004; Wang et al., 2008). Both the active and the precursor form of IL-18 protein were also shown to be highly expressed in human atherosclerotic plaque macrophages. Furthermore, IL-18 mRNA expression was higher in unstable than in asymptomatic plaques (Mallat et al., 2001).

Patients with acute MI also have raised systemic IL-18 concentrations which correlate with increased values of atrial natriuretic peptide (ANP), suggesting a role for IL-18 in ANP induction (Seta et al., 2000; O’Brien et al., 2014). Of note, higher IL-18 concentrations are associated with the development of congestive HF and acute MI in patients with acute coronary syndromes and with increased mortality in elderly patients with HF (O’Brien et al., 2014; Sanchez et al., 2014).

### Interleukin 33

Interleukin 33 (IL-33) is a member of IL-1 cytokine family and was found to be the ligand for the ST2 receptor which belongs to the IL-1 receptor superfamily (Kunes et al., 2010; Altara et al., 2018; Ghali et al., 2018; Segiet et al., 2019a). The ST2 receptor exists as a functionally active transmembrane form (ST2L) and as a soluble “decoy” receptor form (sST2). sST2 is a mechanically induced cardiomyocyte protein that counteracts the anti-hypertrophic action of IL-33 and other IL-33/ST2L-mediated effects (Kunes et al., 2010; Altara et al., 2018; Ghali et al., 2018; Segiet et al., 2019a). There is considerable evidence that sST2 concentration may be used as an indicator of cardiac stress and remodeling in several cardiovascular diseases such as HF, cardiomyopathies, arterial hypertension and aortic stenosis (Ghali et al., 2018). There have been more studies evaluating sST2 than IL-33 because of its higher concentrations and stability (Altara et al., 2018).

IL-33 is constitutively expressed in endothelial cells of both small and large vessels, but not of brain or glomeruli microvessels. Epithelial cells, smooth muscle cells, fibroblasts and keratinocytes also constitutively express IL-33 (Altara et al., 2018; Segiet et al., 2019a). Adipose tissue and endothelial cells of human atherosclerotic plaque also express IL-33 (Demyanets et al., 2011; Ghali et al., 2018). In the heart, it is predominantly expressed in vascular endothelial cells, but it is also present in fibroblasts and cardiomyocytes, having a fivefold higher expression in fibroblasts than in cardiomyocytes (Kunes et al., 2010; Ghali et al., 2018). Of note, endothelial cells appear to be important to translate myocardial pressure overload into a systemic inflammatory response via IL-33 secretion (Chen et al., 2015). Mechanical strain is the main stimulus for the induction of IL-33 expression in the heart, although proinflammatory cytokines such as TNF-α, IL-1β, and IFN-γ also increase its production (Sanada et al., 2007; Kunes et al., 2010; Demyanets et al., 2013). IL-33 is also released during cell necrosis (Demyanets et al., 2013). Angiotensin II (Ang II) also induces IL-33, with both mediators exerting compensatory effects in response to increased cardiac stretch (Kunes et al., 2010).

In contrast to other members of the IL-1 family such as IL-1α, IL-1β and IL-18, IL-33 has predominantly an anti-inflammatory action since it is associated with T-helper type 2 (Th2) immune responses (Kunes et al., 2010). IL-33 exerts cardioprotective effects by reducing cardiomyocyte hypertrophy, cardiomyocyte loss by apoptosis, infarct size, cardiac remodeling and fibrosis (Kunes et al., 2010; Ghali et al., 2018; Segiet et al., 2019a). IL-33 also inhibits the formation of atherosclerotic plaque and reduces angiotensin II-induced ROS and lipid peroxidation products in human cardiomyocytes, an effect that may be attenuated by increased sST2 concentrations (Kunes et al., 2010; Zhang H.F. et al., 2012). However, IL-33 has also been shown to induce proinflammatory cytokines and adhesion molecules in endothelial cells, promoting vascular permeability and angiogenesis and contributing to the early endothelial dysfunction events involved in the development of atherosclerotic lesions (Choi et al., 2009; Demyanets et al., 2011; Pollheimer et al., 2013).

Some studies have shown that IL-33 concentrations are increased, positively correlated with TNF-α and N-terminal-pro-B-type-natriuretic peptide (NT-proBNP) and negatively correlated with LVEF in CHF patients (Zhang H.F. et al., 2012; Xiang et al., 2021). These effects might be due to a reduction of IL-33 bioactivity caused by the increase of sST2 in these patients (Zhang H.F. et al., 2012). Of note, sST2 concentrations are higher in HFrEF than in HFpEF, being a predictor of adverse outcomes in both HF populations (Manzano-Fernandez et al., 2011; Song et al., 2020). In patients with acute worsening of HF, higher sST2 values were also shown to be useful to identify those patients at high-risk of in-hospital death (McCarthy and Januzzi, 2018; Borovac et al., 2020). sST2 concentrations rapidly decreased after hospital admission in AHF patients with uncomplicated short-term follow-up, while AHF patients that died within 6 months showed a significant increase of sST2 values after admission (Boisot et al., 2008; McCarthy and Januzzi, 2018). Furthermore, higher sST2 concentrations appear to be associated with lower diuretic efficiency in patients with AHF and concomitant renal dysfunction (Espriella et al., 2021).

In contrast to the studies describing an increase of IL-33 in human HF, a recent study showed reduced IL-33 concentrations in HFrEF patients when compared to healthy controls, with patients with HF of ischemic etiology presenting lower values than those with non-ischemic etiology (Segiet et al., 2019b). More studies are needed to clarify whether IL-33 is cardioprotective or contributes to chronic inflammation, aggravating the disease (Altara et al., 2018; Segiet et al., 2019b).

### Myeloperoxidase

Myeloperoxidase (MPO) is a haem-containing enzyme mainly secreted by neutrophils and monocytes, under inflammatory conditions, but has also been detected in other cell types such as macrophages (e.g., infiltrating macrophages in atherosclerotic lesions, peritoneal macrophages), CD4^+^ and CD8^+^ lymphocytes, endothelial cells and platelets (Ndrepepa, 2019; Sousa et al., 2019). MPO activity in macrophages probably results from neutrophils endocytosis or MPO internalization, while in endothelial cells, MPO may be endogenously expressed or originate from external sources (Ndrepepa, 2019). MPO uses H_2_O_2_ derived from leukocyte or vascular NADPH oxidases to produce several oxidizing molecules such as hypochlorous acid (HClO), chloramines, tyrosyl radicals and nitrogen dioxides (Ndrepepa, 2019; Sousa et al., 2019). These MPO-derived ROS and reactive nitrogen species (RNS) exert not only bactericidal effects, but also tissue damaging actions in the cardiovascular and renal systems and in the brain (Ndrepepa, 2019; Sousa et al., 2019; Correa et al., 2020). MPO significantly affects vascular tone, endothelial NO availability, being involved in atherogenesis and cardiovascular disease (Csato et al., 2015; Ndrepepa, 2019; Sousa et al., 2019). MPO also contributes to myocardial dysfunction. Experimental studies using MPO knockout mice or an oral MPO inhibitor showed significant less left ventricle dilation and improved left ventricular function in models of myocardial infarction, evidencing a pathophysiological role of MPO in the development of CHF (Askari et al., 2003; Vasilyev et al., 2005; Ali et al., 2016). In humans, CHF patients exhibit higher systemic MPO values, which appear to be associated with worse outcomes (Ng et al., 2006; Tang et al., 2006, 2007). Patients with AHF and CS also show markedly increased circulating MPO values (Reina-Couto et al., 2019b). In acutely decompensated CHF, MPO concentration was also associated with an increased risk for 1-year mortality (Reichlin et al., 2010).

### Inducible Nitric Oxide Synthase

NO is a free radical involved in several physiological processes relevant for cardiovascular regulation, including vasodilation, regulation of cardiac contractility, modulation of sympathetic outflow, smooth muscle cell proliferation, regulation of renal renin release, natriuresis and immune response (Sousa et al., 2019; Cinelli et al., 2020). It is generated by the oxidation of L-arginine to L-citrulline by NO synthases (NOS), a family of enzymes composed of 2 constitutive isoforms, namely neuronal NOS (nNOS or NOS1) and endothelial NOS (eNOS or NOS3), and one inducible isoform (inducible NO synthase, iNOS or NOS2) (Tang et al., 2014; Sousa et al., 2019; Cinelli et al., 2020). iNOS is not normally produced in most cells, being only expressed after induction or stimulation, generally by proinflammatory cytokines (e.g., TNF-α; IL-1β; interferon gamma, IFN-γ) and lipopolysaccharide (Cinelli et al., 2020). After induction, iNOS generates high amounts of NO (100- to 1000-fold more NO than that produced by eNOS). This production persists for many hours until the enzyme is degraded (Soskic et al., 2011; Cinelli et al., 2020). Although these significant amounts of NO are important for the immune response, they can also contribute to toxic effects and to several human diseases, including HF (Soskic et al., 2011; Cinelli et al., 2020).

iNOS was originally discovered in macrophages but has been shown to be expressed in several cell types, including smooth muscle cells, endothelial cells, cardiomyocytes, hepatocytes, neurons, glial cell and astrocytes (Hemmrich et al., 2003; Cinelli et al., 2020). Regarding cardiac function, high concentrations of NO have been shown to exert negative inotropic and chronotropic effects and to reduce the response to β-adrenergic stimulation (Cotton et al., 2002). Importantly, under inflammatory conditions, the reaction of NO with ROS is favored, originating RNS such as peroxynitrite (Pacher et al., 2007). Both excess NO and peroxynitrite can cause deleterious effects in the heart, namely cell apoptosis, contractile dysfunction, irreversible reduction of myocardial oxygen consumption and dysregulation of heart rate and rhythm (Cotton et al., 2002; Kaluski et al., 2006; Pacher et al., 2007). The overexpression of iNOS also appears to contribute to myocardial fibrosis and ventricular hypertrophy (Zhang et al., 2007). iNOS has been detected in the hearts of CHF patients (NYHA classes III-IV), regardless of the etiology (Vejlstrup et al., 1998). Furthermore, it was shown to be uniformly distributed in the left and right ventricles and was primarily located in endothelial and vascular smooth muscle cells of the myocardial vasculature of these patients, being also found, although to a lesser extent, in the cardiomyocyte membrane (Vejlstrup et al., 1998). Infiltrating macrophages also account for iNOS expression in the post-ischemic failing heart (Kingery et al., 2017). Additionally, the activation of iNOS in peripheral vessels of CHF patients (NYHA classes II and III) was shown to be positively associated with systemic BNP concentration and appears to be an independent predictor for worsening HF (Ishibashi et al., 2008). Patients with decompensated CHF were also shown to have increased iNOS expression in peripheral mononuclear cells which was also positively correlated with plasma BNP values (Speranza et al., 2012). As previously mentioned, CS induces a systemic inflammatory response, iNOS activation and excessive production of NO which reduces myocardial contractility, suppresses mitochondrial respiration, attenuates the β-adrenergic inotropic response and induces inadequate systemic vasodilation, leading to systemic hypoperfusion (Kaluski et al., 2006; Shpektor, 2010). The overproduction of peroxynitrite also aggravates myocardial contractile dysfunction (Kaluski et al., 2006; Pacher et al., 2007).

### C-Reactive Protein

C-reactive protein (CRP) is the best studied acute-phase protein. Its synthesis occurs mainly in the liver, being induced by raised IL-6 concentrations under conditions of infection, trauma and other inflammatory states (Huynh et al., 2015; Sheriff et al., 2021). In humans, CRP values markedly increase in the first 72 h after tissue damage, being a sensitive yet non-specific biomarker of inflammation (Huynh et al., 2015; Thiele et al., 2015). CRP is primarily present as a pentamer of five similar polypeptide subunits but can also dissociate into monomers. It is still under debate whether these pentameric and monomeric forms exert different functions. While circulating CRP is pentameric, local deposition of monomeric CRP has been detected in infarcted myocardial tissue, in brain tissue of stroke patients and in the kidney of diabetic patients with severe chronic kidney disease (Thiele et al., 2015; Sheriff et al., 2021).

CRP is a strong chemotaxin/opsonin for macrophages. It binds to phosphorylcholine groups in pathogens and also in non-healthy human cells (e.g., apoptotic, necrotic, energy-depleted, ischemic/hypoxic cells), marking these cells and consequently inducing their phagocytosis through complement activation (Sheriff et al., 2021). The phagocytosis of ischemic/hypoxic cells in the setting of acute myocardial infarction activates IL-6 production, which further induces more CRP, amplifying the immune response. Thus, CRP exacerbates tissue injury and scarring after a cardiovascular event (Griselli et al., 1999; Sheriff et al., 2021). CRP is also present in the myocardium of patients with non-ischemic HF, where it may contribute to myocardial damage through complement system activation and chemotaxis of macrophages (Zimmermann et al., 2009).

CRP can also be produced outside the liver, namely in vascular smooth muscle cells from human coronary arteries, respiratory epithelium, renal cortical tubular cells, neuronal cells, adipocytes and leukocytes (Huynh et al., 2015; Thiele et al., 2015). Noteworthy, CRP appears to be preferentially expressed in diseased vessels, with its mRNA expression being 7–10-fold higher within atherosclerotic plaque compared to the values found in the liver and normal blood vessels (Yasojima et al., 2001; Calabro et al., 2003; Jabs et al., 2003). CRP seems to promote vascular injury by upregulating endothelial adhesion molecules, monocyte chemoattractant protein-1 (MCP-1), endothelin-1 and endothelial plasminogen activator inhibitor 1 (PAI-1), by contributing to the impairment of endothelial NO bioactivity, by increasing low density lipoprotein (LDL) cholesterol uptake by macrophages and triggering LDL cholesterol oxidation and by inducing complement activation (Ridker and Group, 2003; Bassuk et al., 2004).

CRP is an established independent cardiovascular risk factor, with higher CRP values being associated with major cardiovascular events and mortality and showing prognostic significance for risk stratification (Ridker et al., 2003; Koenig et al., 2006; Jan et al., 2008). Since traditional assays of CRP do not effectively detect basal CRP values, assays for the quantification of high-sensitivity CRP (hsCRP) were developed in order to improve risk stratification, with values below 1 mg/L, from 1 to 3 mg/L and higher than 3 mg/L corresponding to low-, moderate- and high-risk groups (Bassuk et al., 2004; Huynh et al., 2015).

Increased CRP values appear to be a predictor for HF development in high-risk populations (Vasan et al., 2003). CRP values higher than 3.23 mg/L are associated with higher HF severity evidenced by lower LVEF, higher NYHA functional classes, higher heart rate and increased prevalence of atrial fibrillation (Anand et al., 2005). In AHF, CRP values are increased by fivefold at admission when compared to the concentrations found in CHF patients. Elevated CRP is also related with worse prognosis in ADHF patients and CRP values above 12 mg/L are associated with increased risk of death and HF readmissions within 3 months (Michelucci et al., 2007; Lourenco et al., 2010).

**FIGURE 1:**
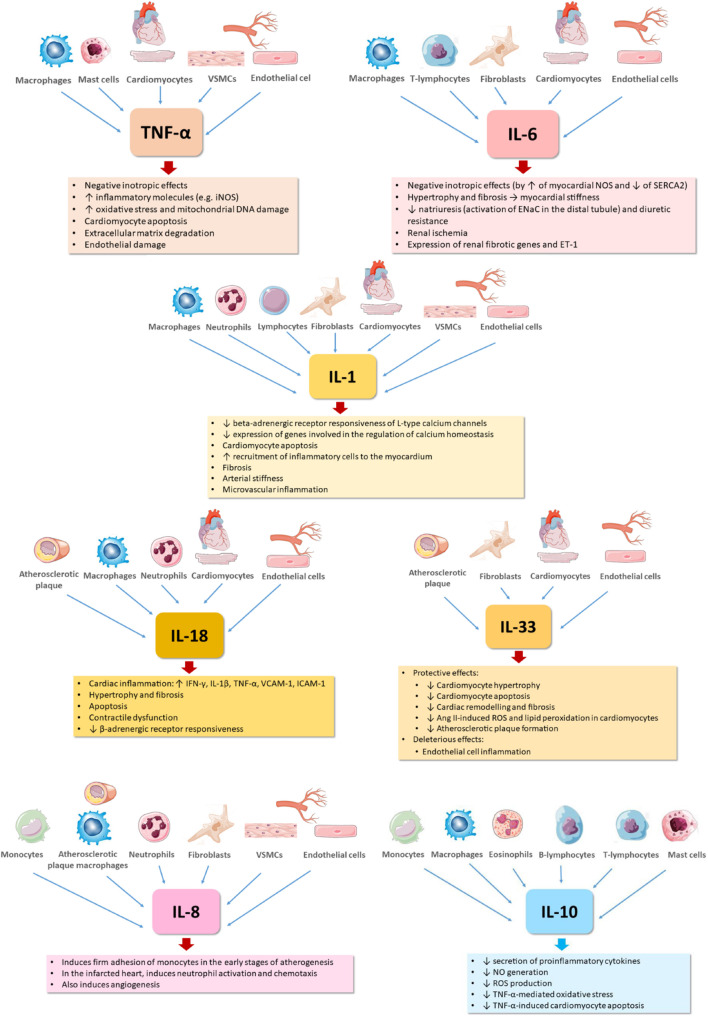
Effects of major cytokines contributing to heart failure pathophysiology. DNA, deoxyribonucleic acid; ENaC, epithelial sodium channel; ET-1, endothelin-1; ICAM-1, intercellular adhesion molecule 1; IFN-γ, interferon gamma; IL-1, interleukin 1; IL-1β, interleukin 1 beta; IL-6, interleukin 6; IL-8, interleukin 8; IL-10, interleukin 10; IL-18, interleukin 18; IL-33, interleukin 33; NO, nitric oxide; NOS, nitric oxide synthase; iNOS, inducible nitric oxide synthase; ROS, reactive oxygen species; SERCA2, sarcoplasmic/endoplasmic reticulum calcium ATPase 2; TNF-α, tumor necrosis factor alpha; VCAM-1, vascular cell adhesion molecule 1; VSMCs, vascular smooth muscle cells.

**FIGURE 2:**
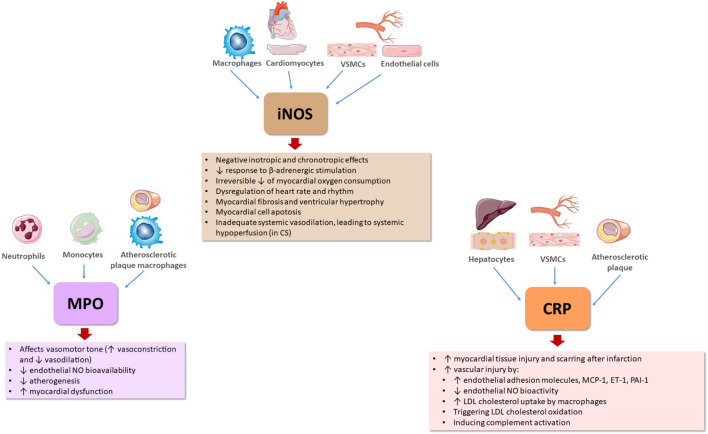
Effects of other important inflammatory mediators contributing to heart failure pathophysiology. CRP, C-reactive protein; CS, cardiogenic shock; ET-1, endothelin-1; iNOS, inducible nitric oxide synthase; LDL, low density lipoprotein; MCP-1, monocyte chemoattractant protein-1; MPO, myeloperoxidase; NO, nitric oxide; NOS, nitric oxide synthase; PAI-1, plasminogen activator inhibitor 1; VSMCs, vascular smooth muscle cells.

The major effects of these inflammatory mediators with relevance for HF pathophysiology are summarized in Figures 1, 2.

## Anti-Inflammatory Strategies in Human Heart Failure

Several direct or indirect anti-inflammatory therapies have been tested in HF patients. Direct therapies include anti-cytokines therapies, prednisone or colchicine, while indirect strategies repurposed drugs that were previously evidenced to exert pleiotropic anti-inflammatory/immunomodulatory effects.

### Anti-cytokine Therapies

Direct anti-inflammatory therapies have yielded conflicting results. Both anti-TNF-α drugs, namely etanercept (Mann et al., 2004) and infliximab (Chung et al., 2003) are now well accepted to be contraindicated in HF, at least in these high-intensity schemes (Adamo et al., 2020; Murphy et al., 2020). The contradictory results between preclinical, observational, preliminary data and the bigger randomized trial might be due to the abolishment of cardioprotective effect of NF-κB, a downstream effector of TNF-α, as well as to the demonstrated infliximab apoptotic and lytic properties (Hori and Yamaguchi, 2013). On the other hand, anti-IL-1 strategy seems more promising. First, anakinra, an IL-1 receptor antagonist, was shown to improve exercise capacity in both small trials in HFrEF (Van Tassell et al., 2012; Abbate et al., 2013) and HFpEF (Van Tassell et al., 2014) but in longer courses in AHF (Van Tassell et al., 2017) it decreased the composite of death or hospitalization for HF; second, canakinumab, a recombinant human monoclonal antibody that targets IL-1β, when added to optimal statin therapy (Ridker et al., 2017), reduced the risk of the composite end point of non-fatal myocardial infarction, non-fatal stroke or cardiovascular death compared with placebo. It showed greater benefit in a dose and hsCRP-dependent fashion, a surrogate biomarker of IL-1 activity, confirming the concept of personalized treatment in HF (Ridker et al., 2018). Nevertheless, although the neutralization of IL-1β or the blockade of IL-1R1 were shown to be protective in human CHF and AHF (Van Tassell et al., 2012; Abbate et al., 2013; Van Tassell et al., 2014, 2016, 2017; Cavalli et al., 2017; Buckley et al., 2018; Everett et al., 2019), circulating IL-1β values are frequently below detection limits, being very difficult to establish a correlation between IL-1β concentration and disease severity (Abbate et al., 2020).

For anti-IL-6 therapy, we still have to wait for major HF trials, with preliminary data in rheumatoid arthritis and in NSTEMI patients showing encouraging results (Kobayashi et al., 2014; Kleveland et al., 2016; Yokoe et al., 2018), as well as for the anti-IL-12/-23 therapy where treatment with ustekinumab in patients with psoriasis was associated with improved echocardiographic measures, lower IL-6 and NT-proBNP (Ikonomidis et al., 2017).

Anti-IL-8 treatment has not been evaluated yet in HF patients, although an antibody targeting IL-8 (BMS-986253) is being tested in clinical trials involving patients with malignant tumors or patients with COVID-19 (Bilusic et al., 2019; Dallos, 2020). Of note, since IL-8 expression is redox regulated (Apostolakis et al., 2009), antioxidants may be useful to therapeutically modulate IL-8 concentrations.

Regarding IL-10 therapy, although its administration was shown to exert cardioprotective effects in animal models (Krishnamurthy et al., 2009; Verma et al., 2012), it has not been tested yet in HF patients.

Therapies targeting IL-18 have been tested in other clinical conditions, but not in HF patients. In patients with type 2 diabetes mellitus or in patients undergoing renal transplantation, trials with a IL-18 neutralizing antibody (GSK1070806) did not show evidence of clinical benefits (McKie et al., 2016; Wlodek et al., 2021). More recently, a clinical trial with a recombinant human IL-18 BP (tadekinig alfa), as well as a short report of a prolonged treatment with this therapy, showed signs of efficacy in patients with a rare systemic autoinflammatory disease (Gabay et al., 2018; Kiltz et al., 2020). With respect to IL-33, it remains to clarify whether it is mainly cardioprotective or contribute to endothelial inflammation, aggravating cardiovascular diseases (Altara et al., 2018; Segiet et al., 2019b). Trials with anti-IL-33 antibodies (etokimab and SAR440340) have been conducted in asthma and atopic dermatitis patients, showing disease improvement with these treatments (Donovan and Hansbro, 2020). Astegolimab, an anti-ST2 antibody that selectively inhibits the IL-33 receptor, was also tested in asthmatics, being able to reduce asthma exacerbation rate (Kelsen et al., 2021). So far, therapies targeting the IL-33/ST2L/sST2 axis have not been tested in human cardiovascular diseases.

### Nitric Oxide Synthases Inhibitors

The role of nitrates and NOS is not unanimous in HF. While in HFrEF, direct donors of NO have its place, at least in patients with optimized treatment or in those not tolerating first line options (Farag et al., 2015) or in ischemic or black patients (Taylor et al., 2004; Real et al., 2018), in AHF, the GALACTIC study failed to demonstrate the improvement in all-cause mortality and re-hospitalization with the early intensive and sustained treatment with nitrates (Kozhuharov et al., 2019).

On the contrary, adjunctive use of NOS inhibition in the setting of CS, based on the theory of overproduction of NO by activated iNOS secondary to systemic inflammatory response (Hochman, 2003), was enthusiastic once again in small single-center trials (Cotter et al., 2000, 2003) with L-NMMA (NG-monomethyl L-arginine), a competitive non-specific NOS inhibitor. However, this was not confirmed in the randomized multi-centre trial SHOCK-2 (Dzavík et al., 2007) nor in TRIUMPH trial (TRIUMPH Investigators et al., 2007), which tested another pan-NOS inhibitor (Kaluski et al., 2006). This lack of clinical benefit might have been caused by the inhibition of constitutive NOS isoforms, which contribute to protective effects on vascular tone and microcirculation, promote ventricular relaxation and prevent platelet adhesion and aggregation (Bailey et al., 2007; TRIUMPH Investigators et al., 2007; Shpektor, 2010). Furthermore, the peroxynitrite-mediated effects are not readily reversible by the acute treatment with NOS inhibitors (Cotton et al., 2002). Nevertheless, newer strategies focusing on the specific inhibition of iNOS or on drugs targeting nitrosative stress should also be tested (Shpektor, 2010).

### Other Anti-inflammatory Therapies

The results of indirect anti-inflammatory therapies have as well to be considered. Statins possess pleiotropic properties, namely antioxidant, angiogenic, immunomodulatory, and also anti-inflammatory effects (Costa et al., 2016) evidenced by the reduction of CRP concentrations independently of lipid reduction (Jain and Ridker, 2005). Again, the observational and *post hoc* analyses data derived from TNT and PROVE-IT studies (Scirica et al., 2006; Khush et al., 2007) and the randomized trials UNIVERSE (Krum et al., 2007), CORONA and GISSI-HF showed neutral effects (Kjekshus et al., 2007; Tavazzi et al., 2008b). Nevertheless, the individualized use of these drugs in patients with atherosclerosis and high LDL-cholesterol could magnify beneficial effects against detrimental inhibition of CoQ10 synthesis that intensifies hypertrophy, especially with high doses of statins in patients with long-term HF (Niazi et al., 2020).

In CHF patients, CRP concentration has also been shown to be reduced by other drugs such as β-blockers (carvedilol) and angiotensin II receptor antagonists (valsartan), but not by the combination of valsartan with angiotensin converting enzyme inhibitors (Anand et al., 2005; Nessler et al., 2013). In addition, in AHF patients, significantly decreased hsCRP concentrations were observed at 30 days after short-term inotropic support (White et al., 2006). However, the putative effect of hsCRP reduction on mortality could not be demonstrated due to the small sample size (Huynh et al., 2015). In acute settings, CRP values need to be rapidly lowered and this cannot be achieved by using CRP-lowering drugs that take several days to influence CRP concentrations (Pepys et al., 2006; Jones et al., 2012; Szalai et al., 2014). This problem can be solved by CRP apheresis, which removes CRP from the blood plasma after myocardial infarction, significantly reducing the infarct area and improving LVEF (Ries et al., 2018, 2019; Mattecka et al., 2019).

Noteworthy, the GISSI-HF study opened new possibilities as the first large scale clinical trial testing omega-3 fatty acids, that when adding 1 g daily to the best medical treatment offered a modest but significant reduction on all-cause mortality and CV hospitalization and proved to be a safe option (Tavazzi et al., 2008a). In a subsequent trial, the reduction seen in inflammatory markers (Moertl et al., 2011), like IL-6 and TNF-α, in the high dose of 4g/d regimen, accompanied by a dose-dependent increase of LVEF and improvement of endothelial function, could justify the outcome benefits. Recent trials, like MESA cohort (Block et al., 2019), showed a significant independent inverse correlation between circulating levels of omega-3 fatty acids, specifically eicosapentaenoic acid (EPA), and the occurrence of HF over a long median follow-up period of 13 years, as well as the ability of plasma EPA concentrations to predict the occurrence of HF in all ethnicities. Also, in the REDUCE-IT trial, very high plasma values of omega-3 fatty acids were associated with a reduction of major CV events (Bhatt et al., 2019) and in the OMEGA REMODEL trial (Heydari et al., 2016), a 6-month treatment with 4 g daily of omega-3 fatty acids on top of the current guideline-based standard of care was associated with a reduction of adverse left ventricular remodeling, myocardial fibrosis and serum biomarkers of systemic inflammation, namely MPO and ST2. Interestingly, EPA is also the precursor of resolvin E1 (RvE1), one of the specialized proresolving mediators actively contributing to resolution of inflammation. Experimental studies have already shown that RvE1, through its receptor ChemR23, exerts a direct protective effect on cardiomyocytes against ischemia-reperfusion injury and limits infarct size when administered intravenously before reperfusion (Keyes et al., 2010; Reina-Couto et al., 2016; Carracedo et al., 2019). Preliminary studies of our group, conducted in patients with AHF and CS, also demonstrated that RvE1 is increased in AHF and CS and correlated with CRP and MPO (Reina-Couto et al., 2019a).

Despite several lines of evidence indicating an association between increased MPO concentrations and HF pathogenesis and severity, the clinical benefit of MPO inhibition in HF patients remains unknown (Ng et al., 2006; Tang et al., 2006, 2007; Reichlin et al., 2010; Ndrepepa, 2019). A clinical trial is currently evaluating the hemodynamic effects of an oral MPO inhibitor, AZD4831, following baseline, resting and exercise testing in HFpEF patients during right heart catheterization (Gan et al., 2019; Nelander et al., 2021).

Serum uric acid (UA) is widely recognized as a biomarker of oxidative stress in several cardiovascular diseases, including HF (Anker et al., 2003). After cellular damage, increased amounts of available xanthine and hypoxanthine can be converted into UA by xanthine oxidase (XO), upregulated itself in cardiovascular disease. Since XO uses oxygen as an electron acceptor, ROS are also generated, contributing to oxidative stress (Zimmet and Hare, 2006; Sousa et al., 2019). Although several studies and meta-analyses have identified elevations of serum UA as an independent marker of poor prognosis in HF patients (Jankowska et al., 2007; Tamariz et al., 2011) and pre-clinical (Stull et al., 2004) and case-control studies (Gotsman et al., 2012), as well as the later OPT-HF study (Hare et al., 2008), showed that XO inhibition was associated with improved survival in HF, the EXACT-HF trial failed to demonstrate a clinical benefit of high-dose allopurinol in HFrEF patients with increased UA concentrations. This lack of benefit might have resulted from the short duration of this study. The authors also suggested that serum UA might be just a marker of disease severity and prognosis and not a target for therapy, even though there was trend toward lower hospitalizations in the allopurinol group of this study. Other possible explanation is that other metabolic pathways, not targeted by the selective inhibition of XO, may be responsible for the nitroso-redox imbalance, as suggested by the unchanged concentrations of MPO in these patients (Tamariz and Hare, 2015).

### Immunomodulators

Other strategies based not only on classic anti-inflammatory schemes, but in a new concept of modulation of inflammation, as shown by the small trials with intravenous immunoglobulin (IVIg), pentoxifylline and thalidomide or the recent repurposed colchicine, could have a role in HF management. IVIg preparations are known to have anti-infectious, anti-inflammatory, and immunomodulatory properties (Nimmerjahn and Ravetch, 2008), but recent prospective, randomized, double-blind, placebo-controlled trial to evaluate the effect of IVIg on systolic cardiac function in adult patients with idiopathic chronic dilated cardiomyopathy (DCM) and parvovirus cardiac B19V persistence (Hazebroek et al., 2021) did not demonstrate improvement of cardiac systolic function or functional capacity beyond standard medical therapy. Importantly, nor even Gullestad et al. (2001a, 2013) could confirm the IVIg protective effects, years later after their study in 2001, in ischemic patients. This treatment is nowadays reserved as an alternative therapy to myocarditis and inflammatory cardiomyopathy in combination with immunoadsorption (Tschope et al., 2021), which still needs validation in multi-center, double blind studies (Bian et al., 2021). The same author, Gullestad et al. (2005), trying the same rationale of beneficial effects of immunomodulation in HF patients, also tested thalidomide. While immunoregulatory (anti-TNF drug, inhibition of neutrophils), as well as matrix stabilizing and antifibrotic properties could contribute to the beneficial effects of thalidomide in HF, a potential risk of harmful effects on the myocardium should also be considered. Our knowledge of its mechanisms of action in chronic HF is limited, awaiting further elucidation in forthcoming studies, as well as the test of newer thalidomide analogs such as lenalidomide, which are more potent and have a more favorable toxicity profile (Aukrust et al., 2007). Interestingly, a meta-analysis of trials with pentoxifylline, a medication with a larger therapeutic index and multiple uses, like limb ischemia or alcoholic hepatitis, seems to suggest a significant nearly fourfold decrease in all-cause mortality in human HF (Champion et al., 2014), even though small trials did not show reduction of mortality. Thus, this xanthinic base with vasodilatatory, anti-inflammatory, antiapoptotic, and rheological properties (Zhang et al., 2004) deserves further exploration in HF. Treatment with colchicine, a well-known alkaloid with potent anti-inflammatory properties, has demonstrated promising results for the secondary prevention of CVD (Webb and Barry, 2020), although it did not provide a benefit in death or HF hospitalization, despite a reduction in inflammatory markers (Deftereos et al., 2014). Thus, the Colchicine in STEMI Patients Study (COVERT-MI), an ongoing confirmative prospective, multicenter, randomized, double-blind trial, is testing whether a short course oral treatment with colchicine versus placebo decreases myocardial injury in patients presenting with STEMI (Bresson et al., 2021), since add-on treatment of coronary artery disease patients already proved to reduce inflammatory biomarkers (Sethuramalingam et al., 2021). The anti-inflammatory effects of methotrexate seem to depend on adenosine receptor stimulation by endogenously generated adenosine (Montesinos et al., 2000). Adenosine is a potent immunomodulatory agent and its receptors activation also induces vasodilation, inhibition of sympathetic neurotransmitter release and induction of ischemic preconditioning, independently of adenosine immunomodulatory effects (Rongen et al., 1997). This could explain why preliminary studies demonstrated cardioprotective effects of raised adenosine concentrations in HF patients (Kitakaze et al., 1998). However, once again, there is still no sufficient data yet to generalize the prevention of incidence of cardiovascular events, demonstrated for rheumatoid arthritis patients, in order to treat patients with coronary heart disease (Sun et al., 2021). Glucocorticoid administration is not recommended routinely in the international guidelines in patients with HF because of its related sodium and fluid retention caused by the stimulation of sodium reabsorption at the level of renal tubules (Ponikowski et al., 2016). On the other hand, few studies have demonstrated that glucocorticoids can enhance natriuresis in HF patients (Liu et al., 2006, 2007; Zhang et al., 2008; Massari et al., 2012), probably by causing vasodilation of the afferent and efferent arterioles and consequently increasing renal blood flow mediated by NO and prostaglandins restricted to kidney bed (De Matteo and May, 1997; de Matteo and May, 1999), as well as by up-regulating ANP synthesis and receptors (Garcia et al., 1985; Lanier-Smith and Currie, 1990). So, larger randomized clinical trials must assure safety and clinical results of COPE-ADHF with enough evidence to promote re-discussion of the main consensus.

Major trials targeting important inflammatory mediators of HF or involving other anti-inflammatory/immunomodulatory strategies in HF are summarized in Tables 1, 2. Most of them were conducted in HFrEF patients, except for the ongoing study with MPO and DHART trials which were specifically designed for HFpEF patients. Contrary to HFrEF, to date, no treatment has been shown to reduce morbidity and mortality in patients with HFpEF (Borlaug, 2020). Although candesartan (Yusuf et al., 2003) and sacubitril/valsartan (Solomon et al., 2020) appear to reduce hospitalizations in patients with LVEF > 40 or 45%, respectively, no randomized clinical trial convincingly reached its primary endpoint. Treating hypervolemia (Adamson et al., 2014) and the mineralocorticoid antagonist spironolactone (Pitt et al., 2014) seemed to produce similar results, with no effect on mortality. On the other hand, nebivolol in SENIORS trial showed promising effects on reduction of cardiovascular mortality but the group of HFpEF considered had LVEF > 35%, including only 15% with a LVEF > 50% (van Veldhuisen et al., 2009). Therefore, the effect of NO-GMPc pathway was also explored but also failed to improve exercise capacity and quality of life (Redfield et al., 2015; Borlaug et al., 2018; Armstrong et al., 2020). Recently, novel evidence emerged strengthening the inflammatory paradigm and reinforcing research for anti-cytokine biological therapy in HFpEF (Paulus and Zile, 2021). For example, higher IL-6 levels were demonstrated to be associated with an increased risk of developing HFpEF (Chia et al., 2021) and circulating levels of TNF-α receptor 2 were shown to be increased in association with the degree of diastolic dysfunction in patients with HFpEF but not HFrEF (Putko et al., 2014). Also, (anti-inflammatory) high density lipoprotein cholesterol/(inflammatory) CRP ratio was shown to be a useful marker for prognostication and correlated with echocardiographic parameters in HFpEF patients (Yano et al., 2021). Moreover, a recent report suggested that the deletion of resolution sensor ALX/FPR2 is associated with the development of diastolic dysfunction related with obesity in mice with HFpEF (Tourki et al., 2020), supporting the concept of non-resolving inflammation in HFpEF (Tourki and Halade, 2021). Besides all this, until now, anti-inflammatory therapies in HFpEF were negative (Murphy et al., 2020), including D-HART2 which failed to improve exercise capacity demonstrated in the pilot study despite lowering CRP and NT-pro-BNP in these patients (Gullestad et al., 2001a; Van Tassell et al., 2018).

**TABLE 1:** Trials targeting major inflammatory mediators of HF.

Target	Trial acronym/registry number and Reference	Study design	Study population	Intervention	Drug mechanism of action	Follow-up	Outcomes
**TNF-α**	Deswal et al., 1999	Randomized double-blind	18 HFrEF patients with NYHA class III (United States) with LVEF of <35%	Intervention groups: single intravenous infusion of 1, 4, or 10 mg/m2 of **etanercept** over 30 min (*n* = 4 for each dose) Placebo group (*n* = 6)	**TNF-α inhibitor** (dimeric recombinant protein fusing the TNF receptor 2 to the Fc region of the human IgG1 antibody)	14 days	- No adverse effects - 4 or 10 mg/m2 of etanercept: ↓ TNF-α, IL 6 ↑ quality of life, 6-min walk test distance and ejection fraction
	Bozkurt et al., 2001	Randomized, double-blind, placebo-controlled	47 HFrEF patients with NYHA class III to IV (United States) with LVEF of <30%	Intervention groups: subcutaneous injections of **etanercept** 5 mg/m2 (*n* = 16) or 12 mg/m2 (*n* = 15) 2x/week for 3 months Placebo group (*n* = 16)	**TNF-α inhibitor** (dimeric recombinant protein fusing the TNF receptor 2 to the Fc region of the human IgG1 antibody)	3 months	- Safe treatment; - Dose-dependent improvement in LV structure, remodeling and function
	Fichtlscherer et al., 2001	Randomized	18 patients with CHF (Germany) with LVEF of <30%	Intervention group (*n* = 13): single dose of subcutaneous injection of 25 mg **etanercept** Control group (*n* = 5)	**TNF-α inhibitor** (dimeric recombinant protein fusing the TNF receptor 2 to the Fc region of the human IgG1 antibody)	7 days	- Improved systemic endothelial vasodilator capacity; - Positive correlation between the increase in ACh-induced-forearm blood flow responses and baseline TNF-α serum values
	**ATTACH** (Anti-TNF Therapy Against Congestive Heart Failure Trial) (Chung et al., 2003)	Randomized double-blind	150 HFrEF patients with stable NYHA class III or IV (United States) with LVEF of ≤35%	Intervention groups: 2-h intravenous infusion of **infliximab** 5 mg/kg (*n* = 50), infliximab 10 mg/kg (*n* = 51) at 0, 2 and 6 weeks Placebo group (*n* = 49)	**TNF-α inhibitor** (Anti-TNF-α, mouse-human chimeric monoclonal antibody)	28 weeks	- No improvement in clinical status - 10 mg/kg infliximab: ↑ risk of death or hospitalization - 5 mg/kg infliximab: ↓ CRP, IL-6; ↑ EF (modestly)
	**RENEWAL** (Randomized Etanercept Worldwide Evaluation): combined data of RENAISSANCE and RECOVER trials in a pre-specified study (Mann et al., 2004)	Randomized double-blind, placebo-controlled	1673 patients with CHF (United Kingdom, Sweden, Germany, Holland, Denmark, Italy, France, Norway, Israel, Australia, New Zealand, United States) with LVEF of <30%	Intervention groups: **etanercept** subcutaneous injection 25 mg 3x weekly (*n* = 308), 25 mg 2x weekly (*n* = 683) Placebo group (*n* = 682)	**TNF-α inhibitor** (dimeric recombinant protein fusing the TNF receptor 2 to the Fc region of the human IgG1 antibody)	24 weeks	- No effects on the rate of death or hospitalization
**IL-1**	**D-HART** (Diastolic Heart failure Anakinra Response Trial) NCT01542502 (Van Tassell et al., 2014)	Randomized, double blind, placebo-controlled, crossover pilot study	12 patients with HFpEF (United States)	SC injection of 100 mg of **anakinra** daily for 14 days and an additional 14 days of placebo or SC injection of placebo daily for 14 days and 100 mg of anakinra for an additional 14 days	**Inhibits IL-1 binding to the IL-1 type I receptor** (recombinant, non-glycosylated form of the endogenous IL-1 receptor antagonist peptide)	28 days	- ↓ systemic inflammatory response - ↑ aerobic exercise capacity of patients with HFpEF and elevated plasma CRP levels
	NCT01936844 (Van Tassell et al., 2016)	Randomized double-blinded placebo-controlled pilot study	30 patients with ADHF (United States) with LVEF of <40%	100 mg **anakinra** twice daily for 3 days followed by once daily for 11 days or matching placebo	**Inhibits IL-1 binding to the IL-1 type I receptor** (recombinant, non-glycosylated form of the endogenous IL-1 receptor antagonist peptide)	14 days	- ↓ systemic inflammatory response in patients with ADHF
	**CANTOS** (Canakinumab anti-Inflammatory Thrombosis Outcome Study) NCT01327846 (Ridker et al., 2017)	Randomized, double-blind trial	10,061 patients with previous MI and hsCRP ≥ 2 mg/L (North America, South America, Europe, Asia, Africa, Australia)	3 doses of **canakinumab** (50 mg, 150 mg, and 300 mg, administered subcutaneously every 3 months)	**IL-1β inhibitor** (monoclonal antibody which binds to human IL-1β, blocking its interaction with IL-1 receptors)	48 months	- ↓ rate of recurrent cardiovascular events than placebo, independent of lipid-level lowering - ↑ incidence of fatal infection than placebo
	**REDHART** (Recently Decompensated Heart Failure Anakinra Response Trial) NCT01936909 (Van Tassell et al., 2017)	Randomized in one of 3 treatment duration arms	60 patients with recently decompensated systolic HF (United States) with LVEF of <50%	1:1:1 ratio to 1 daily subcutaneous injections with **anakinra** 100 mg for 2 weeks, 12 weeks, or placebo	**Inhibits IL-1 binding to the IL-1 type I receptor** (recombinant, non-glycosylated form of the endogenous IL-1 receptor antagonist peptide)	24 weeks	- ↓serum CRP values - ↑ peak VO2 in the group receiving anakinra for 12 weeks.
	**D-HART2 (**Diastolic Heart Failure Anakinra Response Trial 2) NCT02173548 (Van Tassell et al., 2018)	2:1 phase 2, randomized, double-blind, placebo-controlled	31 patients with HFpEF (Sweden)	IL-1 blockade with **anakinra:** 100 mg subcutaneously daily (*n* = 21) or placebo (*n* = 10) for 12 weeks	**Inhibits IL-1 binding to the IL-1 type I receptor** (recombinant, non-glycosylated form of the endogenous IL-1 receptor antagonist peptide)	24 weeks	- ↓ serum hsCRP values - ↓ serum NT-pro-BNP
**IL-6**	Kobayashi et al., 2014	Pilot study	20 women with rheumatoid arthritis (RA) without cardiac symptoms (Japan)	**Tocilizumab** (TCZ; 8 mg/kg IV every 4 weeks) prescribed for patients with RA with an inadequate clinical response to methotrexate	**IL-6 inhibitor** (anti-IL-6 receptor monoclonal antibody which binds to soluble and membrane-bound IL-6 receptors, inhibiting IL-6 signaling)	52 weeks	- ↑increased EF - ↓LVMI associated with disease activity - regression of LV eccentric hypertrophy
	NCT01491074 (Kleveland et al., 2016)	Two-center double-blind, randomized, placebo-controlled phase 2 trial	117 patients with non-ST-elevation myocardial infarction (Norway)	Single dose (intravenous infusion) of the anti-IL-6R antibody **tocilizumab** 280 mg or matching placebo	**IL-6 inhibitor** (anti-IL-6 receptor monoclonal antibody which binds to soluble and membrane-bound IL-6 receptors, inhibiting IL-6 signaling)	3 and 6 months	- ↓ inflammatory response and primarily PCI-related TnT release in NSTEMI patients
	Yokoe et al., 2018		70 patients with RA free of cardiovascular disease	**Tocilizumab** prescribed for patients with active RA - 8 mg/kg of intravenous TCZ every 4 weeks during 24 weeks	**IL-6 inhibitor** (anti-IL-6 receptor monoclonal antibody which binds to soluble and membrane-bound IL-6 receptors, inhibiting IL-6 signaling)	24 weeks	- ↓ NT-pro-BNP levels
	**RESCUE** (Reduction in Inflammation in Patients With Advanced Chronic Renal Disease Utilizing Antibody Mediated IL-6 Inhibition) NCT03926117 (Ridker et al., 2021)	Randomized, double-blind, phase 2 trial (at 40 clinical sites)	264 participants with moderate-to-severe CKD and hsCRP ≥ 2 mg/L (high CV risk) (United States)	66 were randomly assigned to each of the four treatment groups (1:1:1:1) to subcutaneous administration of placebo or **ziltivekimab** 7.5, 15, or 30 mg every 4 weeks	**IL-6 inhibitor** (monoclonal antibody directed against the IL-6 ligand)	24 weeks	- ↓hsCRP values - Ziltivekimab was well tolerated
**MPO**	Hemodynamic Effects of a Novel Myeloperoxidase Inhibitor With Exercise in Heart Failure With Preserved Ejection Fraction - A Randomized, Double-Blind, Placebo Controlled Proof of Principle Study NCT03611153	Randomized, double-blind, placebo controlled proof of principle study	30 HFpEF patients referred to the catheterization laboratory for evaluation of breathlessness or shortness of breath	A single administration dose of 30 mg oral MPO inhibitor (**AZD4831**) or placebo given orally following baseline, resting and exercise testing in patients during right heart catheterization.	Potent and selective **MPO inhibitor**	9–14 days after the study drug dosage	Ongoing study
**CRP**	**CORONA** (Controlled Rosuvastatin Multinational Trial in Heart Failure) (Kjekshus et al., 2007)	Single-blind, randomized, placebo controlled	5,011 II–IV NYHA class ischemic HFrEF patients (371 sites in 19 European countries, Russia, and South Africa) with LVEF of <35%	Intervention group (*n* = 2514): **rosuvastatin** 10 mg daily treatment for at least 3 months or placebo (*n* = 2497)	HMG-CoA reductase inhibitor with **pleiotropic actions** (e.g., antioxidant, anti-inflammatory, improvement of endothelial function)	32.8 months	- ↓ CRP values if CRP > 2.0 mg/L - ↓ hospitalizations for HF - No effect on the composite of cardiovascular-related death, non-fatal MI or stroke;
	**GISSI-HF** (Gruppo Italiano Per Lo Studio Della Sopravvivenza Nell’Insufficienza Cardiaca-Heart Failure) NCT00336336 (Tavazzi et al., 2008b)	Randomized, double-blind, placebo-controlled	4,574 II–IV NYHA class ischemic and dilated cardiomyopathy HFrEF patients (Italy) with mean LVEF of <45%	Intervention group (*n* = 2285): **rosuvastatin** 10 mg daily treatment for at least 3 months or placebo (*n* = 2289)	HMG-CoA reductase inhibitor with **pleiotropic actions** (e.g., antioxidant, anti-inflammatory, improvement of endothelial function)	46.8 months	- ↓ hsCRP values at 3 months - No effect on all-cause death or composite of all-cause death or hospitalization for cardiovascular causes
**NOS**	Cotter et al., 2000	Single-center, preliminary report	11 patients with extensive MI complicated with CS (Israel)	**L-NMMA** - 1 mg/kg bolus and 1 mg/kg/h continuous IV drip for 5 h.	**Non-selective NOS inhibitor**	1–3 months	- No adverse effects - ↑ BP - ↑ Urinary output
	**LINCS** (L-NAME (a NO synthase inhibitor) In the treatment of refractory Cardiogenic Shock) (Cotter et al., 2003)	Single-center, prospective randomized study	30 patients with refractory CS (Israel)	Intervention group (*n* = 15): supportive care in addition to **L-NAME** - 1 mg/kg bolus and 1 mg/kg/h continuous IV drip for 5 h; Control group (*n* = 15): supportive care alone	**Non-selective NOS inhibitor**	4 months	- ↑ BP - ↑ Urinary output - ↓ Time of mechanical ventilation - ↓ Time of intra aortic ballon pump support
	**SHOCK-2** (Should we inhibit nitric Oxide synthase in Cardiogenic Shock 2) (Dzavík et al., 2007)	Multicenter phase II, randomized, placebo-controlled, dose ranging study	79 patients with acute MI complicated by persistent CS despite PCI (United States, Canada, Germany, Israel, Austria, Denmark)	Intervention groups (*n* = 15/15/15/14): **L-NMMA** - 0.15/0.5/1.0/1.5 mg/kg IV bolus and 0.15/0.5/1.0/1.5 mg/kg/h infusion for 5 h; Placebo group (*n* = 20): 0.9% normal saline IV bolus, and 5 h infusion.	**Non-selective NOS inhibitor**	2 h after study initiation (MAP outcome) or 30 days (mortality outcome)	- No adverse effects - ↑ BP at 15 min (modestly) - No effect on BP at 2 h - No effects on glucose and urinary output - No significant differences on mortality at 30 days
	**TRIUMPH** (Tilarginine Acetate Injection in a Randomized International Study in Unstable MI Patients With Cardiogenic Shock) (TRIUMPH Investigators et al., 2007)	International multicenter, randomized, double blind placebo-controlled	398 patients with refractory CS complicating MI despite PCI (eight countries in North America and Europe)	Intervention group (*n* = 206): **Tilarginine (L-NMMA)** - 1 mg/kg bolus and 1 mg/kg/h infusion for 5 h; Placebo group (*n* = 190).	**Non-selective NOS inhibitor**	6 months	- No effect on 30-day all-cause mortality - ↑ SBP at 2 h - No effect on the resolution of shock, on reinfarction, or on renal function.

*ACh, acetylcholine; ADHF, acute decompensated heart failure; BP, blood pressure; CHF, chronic heart failure; CKD, chronic kidney disease; CRF, cardiorespiratory fitness; CRP, C-reactive protein; CS, cardiogenic shock; CV, cardiovascular; EF, ejection fraction; HF, heart failure; HFpEF, Heart failure with preserved ejection fraction; HFrEF, heart failure with reduced ejection fraction; HMG-CoA, β-hydroxy β-methylglutaryl Coenzime A; hsCRP, high sensitive C reactive protein; IL-1, interleukin 1; IL-6, interleukin 6; IL-6R, interleukin 6 receptor; L-NAME, N-Nitro-L-Arginine-Methyl Ester; L-NMMA, N-monomethyl L-arginine; LV, left ventricular; LVMI, left ventricular mass index; MI, myocardial infarction; MPO, myeloperoxidase; NOS, nitric oxide synthase; NSTEMI, non-ST segment elevation myocardial infarction; NT-pro-BNP, N-terminal-pro-B-type Natriuretic Peptide; NYHA, New York Heart Association; PCI, percutaneous coronary intervention; RA, rheumatoid arthritis; SBP, systolic blood pressure; SC, subcutaneous; sICAM-1, soluble intercellular adhesion molecule-1; TCZ, tocilizumab; TNF-α, tumor necrosis factor alpha; TnT, troponin T; VO_2_, volume of oxygen consumption.*

**TABLE 2:** Other anti-inflammatory trials in HF.

Trial acronym/registry number and Reference	Study design	Study population	Intervention	Drug mechanism of action	Follow-up	Outcomes
Gullestad et al., 2001a	Randomized, double-blind, placebo-controlled study	40 II–III NYHA class ischemic and dilated cardiomyopathy HFrEF patients with LVEF of <40% (Norway)	**Intravenous immunoglobulin therapy (IVIG) -** induction therapy (1 daily infusion at 0.4 g/kg for 5 days) and thereafter as monthly infusions (0.4 g/kg) for a total of 5 months or placebo for a total period of 26 weeks (4 weeks after last IVIG or placebo infusion).	**Immunomodulator** (influences the concentration of cytokines and cytokine modulators; neutralizes microbial antigens and autoantibodies; Fc-receptor blockade; complement inactivation)	6 months	- ↑ anti-inflammatory cytokine profile (IL-10, IL-1 receptor antagonist, and soluble tumor necrosis factor receptors) - improvement in clinical status - ↑ LVEF - ↓ N-terminal pro-atrial natriuretic peptide
Sliwa et al., 2002	Prospective, randomized, double-blind, placebo-controlled study	18 IV NYHA class dilated cardiomyopathy HFrEF patients (South Africa) with LVEF of <25%	1-month therapy with **pentoxifylline** (400 mg 3 times daily) (*n* = 9) and placebo (*n* = 9)	**Immunomodulator** (phosphodiesterase inhibitor leading to ↑cAMP and downstream inhibition of proinflammatory mediators)	1 month	- ↓ TNF-α levels and Fas/Apo-1 concentrations - improved symptoms and ↑ LVEF
Sliwa et al., 2004	Single-center, prospective, double-blind, randomized, placebo-controlled	38 II–III NYHA class ischemic HFrEF patients (South Africa) with LVEF of <35%	2 parallel arms: **pentoxifylline** 400 mg TID (*n* = 20) or a matching placebo (*n* = 18) for 6 months in addition to standard therapy	**Immunomodulator** (phosphodiesterase inhibitor leading to ↑cAMP and downstream inhibition of proinflammatory mediators)	6 months	- ↓ in plasma markers of inflammation, prognosis, and apoptosis. - improved symptoms and ↑ LVEF
Gullestad et al., 2005	Double-blind, placebo-controlled study	56 II–III NYHA class ischemic and dilated cardiomyopathy HFrEF patients (Norway) with LVEF of <40%	**Thalidomide** (25 mg QD increasing to 200 mg QD) or placebo and followed up for 12 weeks	**Immunomodulator** (alters the concentration of inflammatory cytokines; downregulates neutrophils)	3 months	- ↓ total neutrophil count and ↑ TNF-α levels - ↓ heart rate - ↑ in LVEF and improvement in left ventricular remodeling with matrix-stabilizing net effect
Gong et al., 2006	Prospective, randomized, placebo-controlled, single-blind study	71 patients with CHF outpatients receiving conventional treatment (China) with LVEF of <35%	Intervention group (*n* = 35): **Methotrexate** 7.5 mg per week for 12 weeks Placebo group (*n* = 36)	Folate analog with **anti-inflammatory properties:** inhibits inflammatory cell proliferation; ↑ extracellular concentrations of adenosine (which exerts anti-inflammatory effects by binding to A_2_ receptors)	12 weeks	- ↓ TNF-α, IL-6, MCP-1, sICAM-1, CRP - ↑ IL-10, soluble IL-1 receptor antagonist - Improved NYHA functional class, 6-min walk test distance and quality of life scores
**GISSI-HF** (Gruppo Italiano Per Lo Studio Della Sopravvivenza Nell’Insufficienza Cardiaca-Heart Failure) NCT00336336 (Tavazzi et al., 2008a)	Randomized, double-blind, placebo-controlled	NYHA functional class II–IV heart failure irrespective of cause and/or LVEF (Italy) with mean LVEF of <45%	Intervention: n-3 polyunsaturated fatty acids (**n-3 PUFA**) 1 g daily (*n* = 3494) vs. placebo (*n* = 3481)	**Precursors of SPMs** (which have proresolving and anti-inflammatory effects). Incorporation of *n*-3-PUFA on the membrane of target cells likely reduces electrical excitability (**anti-arrhythmic** effect).	46.8 months	- ↓ in both all-cause mortality and the composite end point of all-cause mortality and hospitalization for cardiovascular causes in all the predefined subgroups, compared with the placebo group
**METIS** (METhotrexate Therapy on the Physical Capacity of Patients With ISchemic Heart Failure Trial) (Moreira et al., 2009)	Randomized double-blind, placebo-controlled trial	50 patients with ischemic CHF (Brazil) with mean LVEF of <45%	Intervention group (*n* = 25): **Methotrexate** 7.5 mg per week plus folic acid (5 mg/week) for 12 weeks Placebo group (*n* = 25): Placebo plus folic acid (5 mg/week), for 12 weeks	Folate analog with **anti-inflammatory properties:** inhibits inflammatory cell proliferation; ↑ extracellular concentrations of adenosine (which exerts anti-inflammatory effects by binding to A_2_ receptors)	12 weeks	- No effects on CRP - No effects on 6-min walk test distance - Trend toward improved NYHA scores
**COPE-ADHF** (Cardiac Outcome Prevention Effectiveness of Glucocorticoids in Acute Decompensated Heart Failure) (Liu et al., 2014)	Non-blinded randomized	102 patients with ADHF (China) with mean LVEF of <45%	Intervention group: **dexamethasone** (20 mg/d) IV followed by **prednisone** (orally, daily, 1 mg/kg/d with a maximum dose of 60 mg/d) for 7 days and then tapered off in 3 days (*n* = 51); Control group (*n* = 51): standard care	Glucocorticoid receptor agonists that **regulate the transcription of several genes involved in the inflammatory response**. Also ↑ the expression of the receptor for natriuretic peptides (diuretic effect).	30 days	- Safe therapy; - ↓ Serum creatinine - ↑ Diuresis, ↓weight - ↓ CV death at 30 days - Improved dyspnea and clinical status
Deftereos et al., 2014	Single-center, prospective, double-blinded, placebo-controlled study	267 Patients with stable CHF and systolic dysfunction (EF ≤ 40%) (Greece) with mean LVEF of <35%	Intervention group (*n* = 134): oral **colchicine** 0.5 mg twice daily (once daily if weight < 60 kg) for 6 months Placebo group (*n* = 133)	Microtubule inhibitor with anti-inflammatory properties: **inhibits NLRP3 inflammasome activation**; **disruption of leukocyte functions.**	6 months	- Safe use of colchicine; - ↓ hsCRP, IL-6 - No effect on patient functional status, death or hospital stay
**EXACT-HF study** (Xanthine Oxidase Inhibition for Hyperuricemic Heart Failure Patients) (Givertz et al., 2015)	Multi-center, 1:1 randomized, double-blind, placebo-controlled	253 II–IV NYHA class ischemic and dilated cardiomyopathy HFrEF patients (LVEF ≤ 40%) and elevated serum UA levels (≥9.5 mg/dL) (various centers in United States and Canada)	**Allopurinol** was given for 24 weeks starting with 300 mg by mouth once daily for 1 week, and if tolerated, increased to 600 mg daily. Patients unable to tolerate 600 mg were maintained on 300 mg.	Xanthine oxidase inhibitor; besides urate lowering, **↓ oxidative stress and inflammatory mediators.**	6 months	- Failed to improve clinical status, exercise capacity, quality of life, or LVEF at 24 weeks

*ADHF, acute decompensated heart failure; cAMP, cyclic Adenosine Monophosphate; CHF, chronic heart failure; CRP, C-reactive protein; CV, cardiovascular; HFrEF, heart failure with reduced ejection fraction; hsCRP, high sensitive C reactive protein; IL-6, interleukin 6; LVEF, left ventricular ejection fraction; MCP-1, Monocyte chemoattractant Protein-1; NLRP3, NOD-, LRR-and pyrin domain-containing protein 3; NYHA, New York Heart Association; PUFA, polyunsaturated fatty acids; QD, once a day; SPMs, specialized proresolving mediators; sICAM-1, soluble intercellular adhesion molecule-1; TID, three times daily; TNF-α, Tumor necrosis factor alpha.*

## Conclusion

Since the recognition by Levine et al. (1990) of elevated TNF-α in CHF patients, there has been a growing body of evidence on the association of a myriad of cytokines and chemokines with HFrEF but more recently this has also been demonstrated in the entire clinical spectrum of HF like HFpEF and even in AHF and CS (Mann, 2015). The progression of HF was believed to be due, along with neurohormonal activation, to a sustained inflammatory signaling – chronic para-inflammation – a theory that became collectively known as the “cytokine hypothesis” (Seta et al., 1996). Major inflammatory players in HF include TNF-α, IL-1, IL-6, IL-8, IL-10, MPO, iNOS, and CRP, to which experimental and clinical attempts have been directed to target or modulate them. However, the optimal approach seems far to be completed, underscoring the complexity of anti-inflammatory strategies. Although disappointing, negative results do not necessarily argue against the cytokine hypothesis (Dutka et al., 2020). If, in one hand, isolated high intensity anti-TNF-α strategies seem to be rejected, anti-IL-1 and anti-IL-6 therapeutics remain to be explored and individualized. While a new therapeutic strategy such as MPO inhibition is currently being tested, immunomodulators like pentoxyfilline, thalidomide, n-3-PUFAs, glucocorticoids or colchicine, already evaluated in preliminary studies, also deserve further larger research. Trials based on serum CRP and UA have underpinned them as biomarkers and probably not as therapeutic targets. In fact, one of the main conclusions we can get from this cytokine hypothesis history is that probably the future will be to tailor the therapeutics according to an inflammatory profile (Van Linthout and Tschope, 2019) which will require a better knowledge of the players acting on HF immunopathogenesis in order to improve immunomodulatory treatment (Bajaj et al., 2020), or alternatively to promote the resolution of inflammation which remains scarcely studied in human HF (Reina-Couto et al., 2014, 2016; Chiurchiu et al., 2019).

## Author Contributions

MR-C and TS conceived and wrote the manuscript. PP-T, JQ-S, and CS-P wrote the manuscript. TS and AA-T supervised and reviewed the manuscript. All the authors contributed to the article and approved the submitted version.

## Conflict of Interest

The authors declare that the research was conducted in the absence of any commercial or financial relationships that could be construed as a potential conflict of interest.

## Publisher’s Note

All claims expressed in this article are solely those of the authors and do not necessarily represent those of their affiliated organizations, or those of the publisher, the editors and the reviewers. Any product that may be evaluated in this article, or claim that may be made by its manufacturer, is not guaranteed or endorsed by the publisher.
